# The Role of Meta-Emotional Intelligence in Behavioral Rule Knowledge

**DOI:** 10.3390/jintelligence13110136

**Published:** 2025-10-27

**Authors:** Antonella Chifari, Antonella D’Amico, Alessandro Geraci, Luciano Seta, Giuseppe Chiazzese

**Affiliations:** 1 Department of Psychology, Educational Science and Human Movement, University of Palermo, 90128 Palermo, Italy; antonella.damico@unipa.it (A.D.); alessandro.geraci@unipa.it (A.G.); 2 Istituto per le Tecnologie Didattiche, Consiglio Nazionale delle Ricerche, 90146 Palermo, Italy; 3 Department of Law, Economics and Communication, LUMSA University-Libera Università Maria Santissima Assunta, 90145 Palermo, Italy; l.seta@lumsa.it; 4 Institute for Mediterranean Studies, National Research Council, 90145 Palermo, Italy

**Keywords:** emotional intelligence, meta-emotional intelligence, meta-emotional beliefs, positive behaviors intervention and support, assessment, pre-adolescence

## Abstract

Emotional intelligence (EI) and its meta-cognitive counterpart, meta-emotional intelligence (MEI), have increasingly been recognized as key factors in helping students understand, regulate, and reflect on their emotional experiences. MEI expands upon EI by incorporating meta-cognitive beliefs and awareness about one’s own emotional functioning, thereby influencing both emotional regulation and positive behavioral choices. This study examined the relationship between MEI and the knowledge of positive behavioral rules among 198 students aged 9 to 12. Participants completed the IE-ACCME-B, which assesses meta-emotional beliefs, emotional self-conceptualization, and emotional abilities, along with the PBIS-KGVE, a tool developed ad hoc to measure knowledge, generalization, and value-based understanding of school rules. Findings highlight that almost all considered variables are intercorrelated, with meta-emotional beliefs being the best predictor of the students’ knowledge, generalization, and value-based interpretation of behavioral rules. These results suggest the opportunity to establish interventions focused on meta-emotional beliefs to enhance behavioral rule knowledge and foster prosocial development within educational contexts.

## 1. Introduction

Over the past two decades, increasing attention has been given to the role of social, emotional, and behavioral development in shaping not only academic performance but also psychological adjustment and long-term well-being in children and adolescents (Belay 2025; Napolitano et al. 2021; Santos et al. 2023). This shift reflects a broader recognition that cognitive ability alone cannot fully explain individual differences in developmental outcomes, particularly those involving interpersonal relationships, emotional regulation, behavioral adaptation (Soto et al. 2020) and prosocial behavior (Eisenberg and Miller 1987). To this regard, a growing body of research has shown that children and adolescents with higher levels of EI are more likely to engage in prosocial behavior, experience fewer behavioral problems, and adapt more successfully to academic and social challenges (Cao and Chen 2025; Soriano-Sánchez and Jiménez-Vázquez 2023). Specifically, Cao and Chen (2025) recently published a meta-analysis aimed at investigating the relationship between emotional intelligence (EI) and prosocial behavior in children and adolescents, finding a significantly positive association: children and adolescents with higher levels of EI are more likely to engage in prosocial behaviors and experience fewer behavioral problems. In the studies considered by Cao and Chen (2025), prosocial behavior is intended as a range of positive behaviors including positive interactions, altruism, cooperation, empathy, and all “voluntary action intended to benefit another” (Eisenberg et al. 2006; Schroeder and Graziano 2015).

However, especially in the school environment, it may be important to study also positive behavior including actions that are constructive or beneficial but not always directed toward others. For example, a student who studies hard for personal success is showing positive behavior but not necessarily trying to help others. Some examples of positive behaviors in class that are not prosocial include: completing assignments independently, following classroom rules to avoid punishment, and taking neat notes without sharing them.

Positive behaviors are particularly relevant in educational settings, where adequate knowledge of behavioral rules, generalization of learned behaviors, and associated values have been shown to be critical to the harmonic development of both the cognitive and emotional dimensions of students, as well as their adaptation to the classroom and school environment. This adaptation aligns with social norms and shared expectations, often explicitly articulated in the school environment in which the student lives. Expectations are stated with standards of conduct and often convey the characteristics that lead to success both in and out of school (i.e., to be responsible, respectful and to do their best). Clearly stated rules, on the other hand, identify, define, and operationalize concepts of acceptable behavior specific to the classroom setting that are necessary to maintain order and a well-functioning environment (Silveira-Maia et al. 2025). These are all fundamental pillars of the Positive Behavior Intervention and Support (PBIS), a predicted, proactive, and preventative program recognized as one of the most popular evidence-based approach aimed at guiding students in adopting positive behaviors through modeling and positive incentives (Horner et al. 2010; Sugai and Horner 2002). In this framework, positive behaviors are concrete, measurable actions, specified for each setting, that teach “what to do” to meet each expectation/value (*Respect*-*Responsibility*-*Safety*). These positive behaviors include voluntary actions designed to benefit other people but also oneself and one’s workspace (Simonsen et al. 2008). Examples of positive behaviors in a school context are maintaining a tidy workspace, greeting others appropriately, walking with a goal, keeping hands and feet in place, and respecting personal space, among others (Seta et al. 2023).

Considering the second variable examined by Cao and Chen (2025), namely EI, it is important to note that their meta-analysis included only studies investigating trait EI or perceived EI, while none addressed ability EI. This omission is due to the limited availability of reliable assessment tools for measuring ability-based EI in adolescents.

To better understand the implications of these factors, it is essential to trace the historical development of the concept of EI over past decades. Since the 1990s, research on EI has steadily expanded and diversified, giving rise to two main theoretical and methodological approaches: the ability-based and the trait-based models (Ashkanasy and Daus 2005; Joseph and Newman 2010; O’Connor et al. 2019). According to the ability-based approach, originally defined by Mayer and Salovey (1997), EI refers to a set of cognitive abilities involved in perceiving, facilitating, understanding, and managing emotions. In 2016, Mayer, Caruso, and Salovey defined EI as a broad intelligence involved in processing “hot” information and positioning it among other hot intelligences, such as personal and social intelligences, within the second-stratum of the Cattell–Horn–Carroll model (McGrew 2009). Conversely, the trait-based approach, grounded in personality research, defines EI as the affective dimensions of personality located at the lower levels of personality hierarchies (Petrides et al. 2007, 2016), that “essentially concerns people’s perceptions of their emotional world” (Petrides et al. 2016, p. 1).

As regards the measurement approaches, two major methodological approaches are typically employed for the assessment of EI: performance-based tests and self-report instruments (Bru-Luna et al. 2021; O’Connor et al. 2019). Performance tests, based on ability-EI model, are designed to evaluate emotional abilities through emotional problem-solving tasks, whereas self-report instruments, based on trait-EI model, measure typical behaviors in emotion-relevant situations (Ashkanasy and Daus 2005; Bru-Luna et al. 2021; Joseph and Newman 2010; O’Connor et al. 2019). Additionally, a second group of self-report instruments, based on the ability-EI model, have been designed to assess emotional abilities through self-perceptions and assessment, namely self-reported EI or perceived EI (PEI) (Salovey et al. 2002). The main concern around EI theoretical models and assessment involves the convergent, discriminant, and incremental validity of the competing EI measures, which tend to differ across multiple dimensions (Brackett et al. 2006; Brackett and Mayer 2003; Mayer et al. 2008). Self-report instruments depend on individuals’ self-perceptions that may be biased or inaccurate, since emotional abilities may be difficult to self-asses also due to limited social feedback (Mayer et al. 2008). Consequently, self-report and performance-based approaches are likely to reflect distinct facets of emotional functioning (Brackett et al. 2006; Brackett and Mayer 2003). Thus, discrepancies among different measures reflect individual differences in meta-emotional intelligence (MEI) (D’Amico and Geraci 2023), intended as the level of awareness about own emotional abilities. MEI is a multidimensional construct, grounded in both the ability-EI model (Mayer et al. 2016) and metacognition theory (Flavell 1979). Consequently, MEI incorporates emotional abilities and meta-emotional dimensions, such as beliefs and self-perceptions regarding one’s emotional abilities (D’Amico and Geraci 2023). The operationalization of MEI originally involved the IE-ACCME test (D’Amico 2013) a multi-method instrument intended for measuring MEI in preadolescents and adolescents. The IE-ACCME test integrates both self-report and performance measures of EI to assess discrepancies between individuals perceived EI and their actual performance on emotional problem-solving tasks. In addition, it includes a specific scale designed to evaluate individuals’ belief systems about emotions. First empirical results using the IE-ACCME test provided evidence of the importance of MEI for understanding inter- and intra-individual differences in the emotional sphere, adding new insight into the role of EI and MEI in social acceptance by peers (D’Amico and Geraci 2021) or in explaining sex differences in EI (D’Amico and Geraci 2022).

Meta-emotional beliefs, which reflect children’s awareness and evaluation of their own emotional functioning, are assumed to guide behavioral compliance by fostering reflection on the emotional consequences of one’s actions and the appropriateness of responses in social contexts. These reflective mechanisms may therefore represent a key pathway through which MEI supports the internalization of behavioral norms. Building upon this theoretical framework, which encompasses both the domains of positive behavior and MEI, the present study aims to advance the understanding of their reciprocal interrelations. Specifically, it investigates the associations between performance-based ability EI, the reflective and metacognitive processes underlying MEI, and the knowledge, generalization, and value systems associated with positive behavior within an educational context. This analysis can offer valuable insights into the emotional foundations of positive behavior knowledge, thereby informing the development of interventions that promote both emotional and behavioral competencies in educational environments. For these reasons, authors seek to answer the guiding research question: “Do students with high levels of MEI know, generalize, and attribute values to behavioral rules better than those with low levels of MEI?”.

To address this question, the present study assessed the relationship between MEI and students’ knowledge of school behavioral rules by administering two instruments—the IE-ACCME-B (D’Amico et al. 2024) and the PBIS-KGVE (Chifari et al. 2024)—to a sample of students aged 9 to 12, with the aim of identifying which components of MEI best predict rule knowledge, generalization, and value attribution.

## 2. Materials and Methods

### 2.1. Participants

The study involved a total of 237 students from primary and lower secondary schools. A total of 198 students including 103 females and 95 males, aged between 9 and 12 years (M = 11.32, SD = 0.95), successfully completed both the PBIS-KGVE and the IEACME-B questionnaires. At the time of administration, 47 students were attending the fifth year of primary school, and 151 students were attending secondary school: 52 of them were attending the first year and 99 the second year. The students taking part in the study were of low-to-medium socio-economic backgrounds, due to the socio-cultural and economic disadvantages of the two schools located in a peripherical area of the city.

### 2.2. Measures

Data were collected using two tools: the IE-ACCME-B and the PBIS-KGVE, both of which are described in detail below.

#### 2.2.1. The IE-ACCME-B Test

The IE-ACCME-B is a multi-method assessment tool which include both self-report and performance-based measures, designed to evaluate both emotional intelligence (EI) and meta-emotional intelligence (MEI) in children aged 8 to 11 years (D’Amico et al. 2024). It is grounded in the four-branch ability model of EI (i.e., perception, facilitation, understanding, and management of emotions; (Mayer et al. 2016)) and assesses metacognitive dimensions reflecting individuals’ beliefs about emotions and awareness regarding their own emotional functioning, namely MEI (D’Amico and Geraci 2023). The test includes four different scales aimed, respectively, at measuring meta-emotional beliefs, emotional self-concept, ability-EI, and self-assessment of performance. The standardized scores of the last three scales may also be used to derive two further scores for meta-emotional knowledge and meta-emotional self-evaluation. Since the IE-ACCME-B test is currently under the standardization process., in this study, only the simple scores of meta-emotional beliefs, emotional self-concept and ability-EI test have been used.

Meta-emotional Beliefs (CE) scale is a questionnaire that assesses children’s beliefs about the value, role, and utility of emotions (e.g., whether emotions can help with thinking or if complex emotional states are understandable). This scale includes eight items rated on a 4-point Likert scale (0 = Not at all - 3 = A lot). Higher scores reflect a greater emphasis placed by children on the emotional aspects of everyday experiences, as well as a stronger sense of confidence in handling emotional situations. The internal reliability, assessed using Cronbach’s alpha, was α = 0.52.

Emotional Self-Concept (CME) scale is a self-report scale that measures children perceived competence in perceiving, using, understanding, and managing emotions in everyday life (e.g., are you good at understanding people’s emotions by looking at their faces?). This scale is also composed of eight items scored on a 4-point Likert response format (0 = Not at all - 3 = A lot). High scores indicate that children view themselves as capable in the EI domains. The internal reliability, assessed using Cronbach’s alpha, was α = 0.69.

Emotional Ability Test (AE) is a maximum-performance test with 30 items across 8 tasks, each addressing one of the four EI branches (i.e., perception, facilitation, understanding, and managing emotions). Perception of emotions includes face and image tasks, where children evaluate how strongly basic emotions (e.g., joy, fear, anger) are expressed in facial drawings or associated with abstract images. Facilitation of emotions encompasses use and sensation tasks. Children judge how helpful basic emotions are in specific situations and how these emotions relate to physical sensations (e.g., cold, bright). Understanding emotions covers blends and transformation tasks. Children assess how basic emotions combine into complex ones (e.g., jealousy) and track changes in emotional states through short stories. Emotion management includes personal and interpersonal management tasks. Children evaluate how individuals typically respond to emotional challenges and rate the effectiveness of different coping strategies. AE tasks are scored using the general consensus scoring method (Mayer et al. 2002), based on a general consensus sample composed of 364 children (170 males, 194 females). The internal reliability of the overall scale, assessed using Cronbach’s alpha, was α = 0.94. The IE-ACCME-B includes two gender-specific versions (with identical content but adapted pronouns and visuals), and items are visually supported and linguistically simplified for age-appropriate comprehension (D’Amico et al. 2024).

#### 2.2.2. The PBIS-KGVE

The PBIS-KGVE questionnaire is an assessment tool created ad hoc to evaluate knowledge, generalization and associated values of positive behavior of certain behavioral rules that often pose educational challenges in the school setting so that teachers can establish a positive classroom climate based on shared behaviors and values that improve relationships among peers and between peers and referring adults (Chifari et al. 2024). The nine behavioral rules assessed in the questionnaire were selected from a broader set based on a consensus among European schools that participated in the Horizon 2020 ARETE Project, which included among its key objectives the promotion and dissemination of Positive Behavior Intervention and Support (PBIS). The targeted rules are greeting others, walking with a goal, keeping your hands and feet to yourself, keeping your workspace organized, storing your belongings, working independently, standing up for others, helping others (with questions), and letting others be in peace. For each of these behavioral rules, four questions were developed to explore the following areas: Behavioral Knowledge (BK)—assessing students’ knowledge of the rule’s meaning; Behavioral Generalization (BG)—evaluating whether students can apply the rule across different contexts and with various people; Behavioral Values (BV)—measuring students’ recognition of the underlying values (Respect, Responsibility, Safety) associated with each rule; Emotions of Behavior (E)—exploring the emotional responses linked to the enacted behaviors. The questionnaire comprises a total of 36 items, resulting from the combination of the 9 selected rules with the 4 associated questions for each rule. For the purposes of the present study, the relationship between emotions and behavior was explored through Meta-Emotional Beliefs (CE) questionnaire, Emotional Self-Concept (CME) scale, and the Emotional Abilities Test (AE) of the IEACME-B test and the areas BK, BG and BV of PBIS-KGVE questionnaire. The BK area includes items such as “What does ‘greeting others’ mean to you?”, with three single-choice response options, only one of which is correct. The BG subscale presents questions like “Do you always greet everyone you meet in the same way?”, again with three single-choice options. The BV area includes questions such as “Why do you think we greet others?”, with four multiple-choice options, where students may select one or more answers depending on the values underlying the behavioral rule. The correct answer is the one that identifies all the values underlying the behavior.

The internal consistency for each area was as follows: BK (Cronbach’s α = 0.52), BG (Cronbach’s α = 0.55), and BV (Cronbach’s α = 0.79). Scores for each area are calculated by adding the points assigned for each correct answer (1 point), with individual scores ranging from 0 to 9. The total score of the questionnaire (hereinafter BKGV) was obtained by summing only the BK, BG, and BV scores, resulting in a possible range from 0 to 27.

### 2.3. Data Analysis

All analyses were conducted using R 12.1. Descriptive statistics (means, standard deviations) were calculated for all study variables. Pearson correlation analyses were performed to examine the associations between the dimensions of emotional and meta-emotional intelligence (CE, CME, AE) and behavioral knowledge, generalization and associated values of positive behavior outcomes (BK, BG, BV) and their total score, (BKGV).

Multiple regression analysis was used to test the predictive contribution of CE, CME, and AE on BKGV, with assumptions of normality, homoscedasticity, and multicollinearity explicitly checked and met. More specifically, BKGV score was entered as criterion, while CE, CME, and AE were entered as predictors.

Finally, a dominance analysis was performed to assess the relative importance of CE, CME, and AE in predicting BKGV. General dominance weights were computed by averaging incremental R^2^ values across all combinations of predictors at increasing levels of model complexity. This allowed us to identify the most consistent and robust predictor of BKGV across different model specifications.

### 2.4. Procedure

The questionnaires were administered collectively in classroom settings. The administration was conducted by researchers, under the supervision of teachers, using two online platforms: Google Forms and LimeSurvey. Specifically, the IE-ACCME-B test was administered through Google Forms. Students accessed the questionnaire via a direct link and completed it within the same session, also lasting about 60 min. While the PBIS-KGVE was administered first via LimeSurvey. Each student was assigned an anonymous login code to access the platform. After reading the instructions, students completed the questionnaire individually in a single session lasting approximately 60 min.

## 3. Results

Correlation analysis was conducted to explore the associations among the total score of the PBIS-KGVE (BKGV), its three components Behavioral Knowledge (BK), Behavioral Generalization (BG) and Behavorial Values (BG), and the three IE-ACCME-B test scores, namely Meta-emotional Beliefs (CE), Emotional Self-Concept (CME), and Emotional Ability Test (AE) scores. These relationships are visually summarized in Table 1, which presents mean and standard deviations along with a correlation matrix highlighting the strength and significance of the associations between the IE-ACCME-B test scales and the PBIS-KGVE dimensions. The BKGV score was significantly and positively correlated with meta-emotional beliefs (CE: *r* = 0.34, *p* < 0.01), emotional self-concept (CME: *r* = 0.27, *p* < 0.01), and emotional abilities (AE: *r* = 0.25, *p* < 0.01). Significant correlations also emerged between the behavioral subcomponents and the IE-ACCME-B test scales. Behavioral Knowledge (BK) scores were positively correlated with CE (r = 0.16, *p* < 0.05), CME (*r* = 0.18, *p* < 0.05), and AE (*r* = 0.21; *p* < 0.01). Behavioral Generalization (BG) scores were also associated with CE (*r* = 0.35, *p* < 0.01), CME (*r* = 0.28, *p* < 0.01), and AE (*r* = 0.34, *p* < 0.01. Lastly, Behavioral Values (BV) scores were positively correlated with CE (*r* = 0.25, *p* < 0.01) and CME (*r* = 0.17, *p* < 0.05). These results underscore a consistent, moderate-to-strong positive relationship between the several aspects of emotional and meta-emotional intelligence dimensions and children’s knowledge, generalization, and internalization of positive behavioral rules.

A multiple regression model was then tested to examine which aspect of emotional and meta-emotional intelligence was the best predictor of BKGV (see Table 2). Thus, the three IE-ACCME-B scale scores were entered as predictors while the BKGV score was the criterion. The model met all key assumptions: residuals were normally distributed (*W* = 0.989, *p* = 0.135), variance was homogeneous (Breusch–Pagan *p* = 0.169), and multicollinearity was not present (all VIFs < 2). The model was statistically significant [*F* (3, 194) = 14.2, *p* < 0.001] and explained 17% of the variance in PBIS total scores (Adjusted R^2^ = 0.17). Among the predictors, only CE (*B* = 2.44, *p* < 0.01) and AE (*B* = 17.67, *p* < 0.001) contributed significantly, while CME did not.

A dominance analysis further supported the significant role of CE, which yielded the greatest average increase in explained variance when entered the model across all combinations of predictors. A dominance analysis was conducted to compare the relative contributions of three IE-ACCME test scales (CE, CME, and AE) to the prediction of BKGV. General dominance weights were estimated by averaging incremental R^2^ values across all model combinations at increasing levels of complexity (i.e., with zero, one, or two additional predictors). The analysis revealed that meta-emotional beliefs (CE) contributed the most consistently across all levels of the model (Level 0: R^2^ = 0.11; Level 1: R^2^ = 0.08; Level 2: R^2^ = 0.04), followed by emotional abilities (AE) (Level 0: R^2^ = 0.06; Level 1: R^2^ = 0.06; Level 2: R^2^ = 0.06). The contribution of emotional self-concept (CME) was notably smaller (Level 0: R^2^ = 0.07; Level 1: R^2^ = 0.04; Level 2: R^2^ = 0.01). These results reinforce the significant role of CE in explaining variation in behavioral learning outcomes, both independently and in combination with other emotional variables.

## 4. Discussion

The present study aimed to investigate the relationship between EI, MEI and students’ knowledge, generalization, and value attribution of behavioral school rules. The understanding of this relationship is particularly relevant in educational contexts, where the correct internalization of positive behavioral rules has proven to be of fundamental importance in promoting students’ well-being (Hassani 2024).

Consistent with previous studies demonstrating more, in general, the role of emotional intelligence in predicting social and academic adjustment (Cao and Chen 2025; Soriano-Sánchez and Jiménez-Vázquez 2023), results reveal a moderate yet robust association between meta-emotional beliefs, emotional abilities, emotional self-concept and students’ knowledge of positive behavioral rules. Notably, meta-emotional beliefs, reflecting children’s metacognitive understanding of the nature and utility of emotions, emerged as the strongest and most consistent predictor of positive behavioral knowledge across correlation, regression, and dominance analyses. These findings contribute to the growing body of literature on how EI and MEI support not only interpersonal functioning and well-being but also the acquisition of prosocial and norm-based behavioral repertoires essential to school adaptation (Cao and Chen 2025; D’Amico and Geraci 2021; Denham and Burton 2003; Soriano-Sánchez and Jiménez-Vázquez 2023).

Interestingly, emotional self-concept, which reflects students’ perceived competence in handling emotions, showed weaker predictive value. This may indicate that self-perceptions of emotional efficacy, while relevant, do not directly translate into deeper understanding or generalization of positive behaviors, due to their subjective and self-evaluative nature. Future research could further investigate this dissociation between perceived competence and actual behavioral competence, especially in young populations where self-concept may be under development.

Concerning EI abilities, they significantly correlated to behavioral knowledge, and this indicate that ability and performance-based EI is more important than perceived EI in predicting the knowledge, generalization and associated values of positive behavior. However, regression analysis showed ability EI to be a weaker predictor than meta-emotional beliefs. This may indicate that the reflective and evaluative dimensions appear more crucial in supporting the generalization and value-based understanding of behavior. The dominance analysis further reinforces this interpretation, showing that CE consistently contributed the largest share of explained variance in total behavioral knowledge. A recent study by Kisley et al. (2024) examined different studies and scales exploring emotion beliefs. Unfortunately, the study did not employ a similar metaemotional belief scale included in the present study. However, the authors claim that emotion beliefs are shaped by early experiences, cultural norms, and interpersonal feedback, and they have downstream effects on behavior, including conformity to social rules and expectations. In this sense, we could argue that students owning a belief system about emotion that value the emotional experience in everyday life—as measured by the meta-emotional beliefs scale—are more likely to understand and learn positive behaviors and to apply them in everyday life.

Moreover, our results indicate that children’s beliefs about the meaning and usefulness of emotions may play a more significant role in the acquisition of behavioral rules than ability EI. For this reason, educational interventions should go beyond the empowerment of emotional abilities and competencies and include opportunities for reflection on the role and value of emotions in everyday life, in interpersonal relationships and in the internalization of social rules (Denham and Burton 2003; Eisenberg et al. 2002). According to the findings, considering the relevance of individual differences in meta-emotional functioning can be useful in enhancing the understanding of the mechanisms that may influence students’ achievement of positive behaviors, adding a new component to the analysis of the processes through which students’ beliefs, knowledge, and motivations are translated into acts and behavior in line with the social expectations of the school context in which they live (Collie 2022).

These findings carry important implications for educational practice. Interventions aimed at promoting positive behavior in schools, such as PBIS programs, could be enhanced by explicitly incorporating meta-emotional training, particularly around beliefs about emotions and their functional role in guiding behavior. Training children not only in how to regulate their emotions but also in why and when emotional regulation is important may facilitate greater internalization of school norms and promote positive behavior.

## 5. Limitations and Future Directions

The present study is characterized by several limitations. First, the cross-sectional and correlational design prevents any causal inference; therefore, longitudinal or experimental studies are needed to explore whether higher emotional and meta-emotional intelligence leads to increased behavioral knowledge over time or vice versa. Second, the study of positive behaviors relied exclusively on the knowledge of rules, their generalization, and the associated values, without incorporating direct observations on the actual behaviors adopted by students. This represents an important limitation, as it prevents us from verifying whether meta-emotional beliefs translate into actual behavioral enactment. Future research should therefore adopt multi-method approaches, integrating behavioral observations and teacher/peer ratings to provide a more comprehensive picture. Additionally, the internal reliability of some subscales (e.g., CE in the IE-ACCME-B; BK and BG in the PBIS-KGVE) was modest (α ranging from 0.52 to 0.55). This is another limitation, as such low reliability may have attenuated the observed relationships and limited the validity of the conclusions, suggesting the need for refinement of tools and further psychometric validation. Another limitation concerns the generalizability of our findings. The sample was drawn from schools located in socio-economically disadvantaged areas, which may restrict the extent to which the results can be extended to students from different socio-economic or cultural contexts. Replicating the study in schools with more diverse socio-economic profiles will be essential to confirm the robustness and external validity of the observed associations.

Finally, although the variance explained by the model was statistically significant, it remained moderate, indicating that other factors beyond emotional and meta-emotional intelligence also contribute to students’ behavioral rule knowledge. These may include contextual variables such as family climate, classroom modeling, peer influence, and the clarity and consistency of school-wide expectations.

Future research should explore how meta-emotional beliefs develop across childhood and how they interact with contextual variables such as peer relationships, family emotional culture, and classroom climate. In addition, it would be valuable to examine potential mediating variables such as students’ motivation and emotion regulation strategies to better understand the mechanisms through which meta-emotional beliefs influence the acquisition and internalization of behavioral knowledge.

Longitudinal and experimental studies could clarify whether meta-emotional beliefs have a causal role in behavioral rule knowledge, and whether it can be strengthened through targeted interventions. Additionally, expanding the sample to include diverse age groups and socio-cultural backgrounds would improve generalizability and help identify potential moderators of the emotional and meta-emotional intelligence and behavior relationship. It may also be valuable to examine how teachers and caregivers’ emotional beliefs and behaviors influence children’s meta-emotional beliefs and behavioral learning, providing a more ecological understanding of emotional and social development in educational contexts.

Last, since the IE-ACCME-B test is still in the standardization phase, we could not examine the role in the prediction of positive behaviors of two important metaemotional dimensions that it will be possible to compute using the IE-ACCME-B test: the meta-emotional knowledge and meta-emotional self-evaluation. These two variables, which measure the discrepancies between self-assessment (CME and AP, not used in this study) and performance test scores (AE), provide insight into individuals’ metacognitive awareness of their EI and will open important new insights about the relationship with positive behaviors. This investigation will be the first future perspective of the present work.

## 6. Conclusions

This study advances our understanding of the mechanisms by which emotional and meta-emotional intelligence, particularly meta-emotional beliefs, plays a fundamental role in the knowledge of positive behavioral rules among school-aged children. In doing so, the study contributes to fill a critical gap in the literature by linking emotional and meta-emotional intelligence not only to emotional outcomes but also to the cognitive and behavioral processes involved in the knowledge of social norms, with significant implications for both educational practice and developmental theory (Knoff 2012). Our findings underscore the need for educational programs to move beyond purely skill-based approaches to meta-emotional learning that include reflective and metacognitive components that foster students’ appraisal of emotions as meaningful tools for navigating social norms. Positive Behavioral Interventions and Supports (PBIS) programs could be further strengthened by explicitly integrating meta-emotional training. Encouraging students to reflect on why emotions matter for behavior and providing teachers with strategies to promote meta-emotional awareness, may enhance not only students’ emotional understanding but also their ability to internalize and enact prosocial behaviors in school settings. Ultimately, promoting meta-emotional belief may serve as a catalyst for aligning students’ emotional development with the expectations of the educational context, thereby enhancing both social adaptation and academic success.

## Figures and Tables

**Table 1:** Descriptive statistics and correlations analysis results.

	Mean	SD	1	2	3	4	5	6	7
1. BKGV	14.22	4.10	-						
2. BK	6.24	1.79	0.74 **	-					
3. BG	6.68	1.77	0.73 **	0.42 **	-				
4. BV	2.01	2.11	0.77 **	0.28 **	0.33 **	-			
5. CE	1.80	0.45	0.34 **	0.16 *	0.35 **	0.25 **	-		
6. CME	1.82	0.51	0.27 **	0.18 *	0.28 **	0.17 *	0.64 **	-	
7. AE	0.46	0.06	0.25 **	0.21 **	0.34 **	0.07	0.01	−0.01	-

Note. AE = Emotional Ability Test; CE = Metaemotional Beliefs Scale; CME = Emotional Self-Concept Scale; BKGV = Total score of PBIS-KGVE; BG = Behavioral Generalization; BK = Behavioral Knowledge; BV = Behavioral Values. * *p* < 0.05, ** *p* < 0.01.

**Table 2:** Multiple regression analysis using BKGV as the criterion.

Predictor	B	β	SE	*t*	*p*
(Intercept)	0.24		2.41	0.10	0.922
CE	2.44	0.27	0.77	3.19	0.002 **
CME	0.81	0.10	0.68	1.19	0.238
AE	17.67	0.25	4.61	3.83	<0.001 ***

Note. AE = Emotional Ability Test; CE = Metaemotional Beliefs Scale; CME = Emotional Self-Concept Scale; ** *p* < 0.01,*** *p* < 0.001.

## Data Availability

The raw data supporting the conclusions of this article will be made available by the authors on request.
